# Hand Hygiene Social Norms Among Healthcare Workers During Early COVID-19: Results of a Global Survey

**DOI:** 10.3389/ijph.2022.1604981

**Published:** 2022-11-24

**Authors:** Giorgia Gon, Aron Szekely, Hattie Lowe, Marco Tosi

**Affiliations:** ^1^ London School Hygiene and Tropical Medicine, London, United Kingdom; ^2^ Collegio Carlo Alberto, Turin, Italy; ^3^ Institute of Cognitive Sciences and Technologies, Italian National Research Council, Rome, Italy; ^4^ Institute for Global Health, University College London, London, United Kingdom; ^5^ Department of Statistical Sciences, University of Padua, Padova, Italy

**Keywords:** healthcare workers, COVID-19, survey, hand hygiene, social norms

## Abstract

**Objectives:** Poor hand hygiene among healthcare workers is an important driver of infectious disease transmission. Although social norms are considered a key determinant of hand hygiene behaviour, little is known about them among healthcare workers. This study describes hand hygiene social norms among health workers, assesses their predictors, and tests if social expectations increased during the early stages of COVID-19.

**Methods:** We conducted a cross-sectional survey of healthcare workers from 77 countries (*n* = 1,233) from April to August 2020 assessing healthcare workers’ hand hygiene social expectations, personal normative beliefs, punishment and reward, and demographic factors. Linear regressions and hierarchical linear modelling were used to analyse the responses.

**Results:** We find high social expectations, personal beliefs, punishment, and rewards. Doctors tend to have lower social expectations than other occupation groups (e.g., nurses/midwives) and older respondents have higher social expectations. Social expectations increased during our survey, which may have been driven by COVID-19.

**Conclusion:** Our findings suggest that hand hygiene social norms are strong among healthcare workers with variation across occupation and age; their strength increased during the COVID-19 pandemic. These have implications for behaviour change in healthcare environments that could leverage more norm-targeting interventions.

## Introduction

### Background

Infection prevention in healthcare settings is essential for ensuring patient safety and practicing hand hygiene at key times during patient care is considered the most important way to prevent infection transmission by health workers (1). Social norms are argued to be a key motivation for hand hygiene behaviour (2, 3). Yet there is little quantitative evidence about hand hygiene social norms in the healthcare environment, where the behaviour is expected hundreds of times a day by each health worker and at specific times related to patient interaction. Moreover, hand hygiene has been a key strategy for responding to the COVID-19 pandemic (4) and some evidence suggests that COVID-19 increased hand hygiene norms among the general population (5, 6). Yet evidence is lacking for healthcare workers. Our contribution to filling these gaps is two-fold. First, we investigate social norms of hand hygiene among health workers. Second, we do this in many different countries with variations in the incidence of COVID-19 contagions, policies, and hand hygiene norms.

Social norms are powerful means of shaping behaviour (7–9). They are informal behavioural rules that specify what actions should be performed within a given social context (10, 11) and are a mechanism through which culture is maintained and changed. In the context of healthcare, they can support the creation a culture of safety—a pillar of the WHO strategy for improving infection prevention within health facilities (1).

We adopt the framework of Bicchieri (10, 12) which argues that social norms are supported by two types of expectations: *empirical expectations* and *normative expectations* (hereafter we use “social expectations” to refer to the collection of both empirical and normative expectations). Empirical expectations are people’s beliefs about how other people *behave* while normative expectations are beliefs about how other people think that people *should behave*. The latter have a normative element while the former do not. Behaviour is said to be supported by norms when people follow a behavioural rule due to empirical and normative expectations. These expectations are different to personal normative beliefs: one’s belief about appropriate behaviour which are first-order beliefs. Social reward and punishment also play key roles in norm emergence and maintenance.

Social norms are influenced by the reference group and by the environment. Robust results across multiple studies suggest that hand hygiene compliance varies with profession, with higher compliance shown among nurses compared to doctors (13, 14) and some evidence suggesting that hand hygiene compliance is lower in unpredictable and busy wards than in quiet wards (14). For these reasons, we study profession, seniority, and ward business.

Our study focuses on hand hygiene (either hand washing with soap and water or hand rubbing with alcohol-based gel) before patient interactions instead of after touching or exposure to body fluids. While both before and after are key times, hand hygiene compliance is substantially lower in the former than the latter (13, 15). This may be because after patient interaction hand hygiene can be driven by instinctive emotional drives whilst before touching a patient needs to be supported by other motivations leading to this discrepancy.

### Objectives

Using the first global survey on social expectations (empirical expectations and normative expectations) personal normative beliefs, rewards, and punishment of hand hygiene among healthcare workers, we aim to:

1. Describe their social expectations, punishment appropriateness, reward experience, and personal normative beliefs of hand hygiene.
2. Assess selected factors associated with social expectations of hand hygiene.
3. Assess if social expectations concerning hand hygiene increased during the early stages of the COVID-19 pandemic, accounting for potential confounders.

## Methods

This study relies on a cross-sectional design and cross-national sample including health workers from 77 countries. We used the STROBE checklist for cross-sectional studies to structure and report our study (16).

### Survey Sample

We used a non-probability online survey. A short questionnaire (administered through Google Forms or Kobotoolbox for China only) was delivered to healthcare workers worldwide through international networks and e-mail lists (e.g., *via* WHO Infection Prevention and Control Unit, WHO WASH in HFC group, FIGO—International Federation of Gynecology and Obstetrics). Questionnaire distribution was also shared with experts in social norms and hand hygiene and through their social networks. For example, on Twitter the questionnaire was tagged to the WHO #SafeHands campaign. There was an element of snow-ball distribution in that participants were asked to send the questionnaire to at least three of their colleagues. We chose to spread our survey in multiple ways because this is a hard-to-reach population while only focusing on social media would reduce the probability that healthcare workers in some countries could participate. Participation in the questionnaire was voluntary and there were no fees or compensation associated with it. The questionnaire was circulated for the first time on the 8th of April 2020 and data collection finished on the 13th of August 2020.

Healthcare workers were eligible to complete the questionnaire if they had been performing clinical duties (occasionally or regularly) in the last 2 months. Participants self-administered the questionnaire on a laptop or mobile phone with an internet connection. The questionnaire was available in English, as well as other 19 languages widely spoken worldwide. No discomfort was anticipated for the participants whilst completing the questionnaire as the questions are not sensitive in nature. The English version of the survey can be found in the Supplementary Material.

### Survey Content

The questionnaire focused on hand hygiene before patient contact. By hand hygiene we specified either hand washing with soap and water or hand rubbing with alcohol-based gel (see Supplementary Material). To maximise responses, our questionnaire included only ten questions. Seven were norm-related (see Table 1) and based on a prior tool (17) that has been used for measuring social norms of community sanitation in low resource settings (18) and healthcare hand hygiene behaviour in Tanzania (19). The questionnaire was completely anonymous and no individual data was collected other than respondents’ gender, profession, and age-group.

**TABLE 1:** Survey content: social expectations, rewards, punishment, and personal normative beliefs items (77 countries, 2020).

Construct	Question	Answer option		
Personal Normative Beliefs (PNB)	Do you think **you should** rub/wash your hands before touching a patient?	No, never (1)	Yes, usually (2)	Yes, always (3)
Normative Expectations (NE)	We recently asked the previous question to many other healthcare workers in your area. On average out of 10, how many healthcare workers thought **they should** always rub/wash hands before touching a patient?	(0–10)		
Empirical Expectations (EE)	Think about healthcare workers in your area. On average out of 10, how many do you think **actually do** always rub/wash hands before touching a patient?	(0–10)		
Empirical Expectations when ward is busy (EE busy)	Think about healthcare workers in your area. On average out of 10, how many do you think **actually do** always rub/wash hands before touching each patient **when caring for several patients during a busy shift**?	(0–10)		
Empirical Expectations for senior as the target group (EE senior)	Think about clinical **senior members** in your area. Out of 10, how many do you think **actually do** always rub/wash hands before touching a patient?	(0–10)		
Punishment appropriateness (Punish)	Is it appropriate for a supervisor to challenge a colleague who did not rub/wash her/his hands before touching a patient?	No, never (1)	Yes, usually (2)	Yes, always (3)
Reward experience (Reward)	In the past 2 weeks, have you experienced any support or open promotion of hand hygiene?	No, never (1)	Yes, once (2)	Yes, multiple times (3)

### Other Data

Country-level data on GDP per capita (Adjusted to PPP) were extracted from the “World Bank International Comparison Program database.” We also gathered data on population size from the United Nations “2019 Revision of World Population Prospects” and on the stringency of government interventions from the Oxford COVID-19 Government Response Tracker (20). Macro-data were linked with survey data using the date of each questionnaire completion. COVID-19 case data, used for robustness checks, was taken from Our World in Data (21).

### Analytic Strategy

Our analysis proceeds in three steps.

1. Summarise the results of the questionnaire using linear regressions with cluster-robust standard errors at the country level. Missing values were described and excluded from the models. To study the variance partitioning (within or between clusters) we estimate the intra-cluster correlation for social expectations and personal beliefs using multilevel linear regression null models with country-level random intercepts and cluster robust standard errors at the country level. This allows us to check if our results are consistent with previous research which has found that most variation in hand hygiene behaviour among healthcare workers is within clusters (22).
2. To assess the associations among social expectations, personal normative beliefs, and individual factors we use multilevel linear regression models with random intercepts at the country level and cluster robust standard errors at the country level. This analysis helps us understand how social expectations are related to the other factors—personal normative beliefs, rewards, and punishment—that are typically considered when understanding social norms.
3. To test if social expectations and personal normative beliefs increased as COVID-19 progressed during the study period (operationalised as date since start of data collection), after accounting for potential confounders (e.g., personal normative beliefs, reward, punishment, age category, occupation), we built multilevel linear regression models with random intercepts at the country level. Depending on the specification, we include individual and macro-level control variables. We further check the robustness of our estimates by estimating a random coefficient for date, controlling for population size, government policy stringency, and estimate standard errors without clustering at the country level.

We use date as the key predictor in step 3 because we posit that it captures a general exposure to COVID-19, with later dates indicating more exposure. Specifically, date likely captures a mix of individual’s direct responses to the pandemic, social influence, governmental policy, and media information. Our key assumption here is that a longer duration since the start of the survey indicates a longer (or greater) exposure to the sum of the pandemic’s consequences. This is likely fulfilled since at the time that our data were collected (April to August 2020) COVID-19 and its consequences were still increasing, or at least not decreasing. While this approach means we cannot disentangle pathways driving change, doing so is beyond the scope of this study. Indeed, our aim is to identify if there is evidence for an association for the sum of the pandemic’s effects and social expectations. An alternative approach, including COVID-19 deaths and cases in the models, would not help specify pathways since both covary with governmental measures, social influence, and media messaging, and particularly at the start of the pandemic, there were fundamental data issues with deaths and cases (e.g., due to differences in COVID-19 monitoring and reporting).

Three of our outcomes, personal normative beliefs, punishment appropriateness, and reward experience, are ordinal. For simplicity and ease of interpretation, we analyse these outcomes as continuous in the main text. In the Supplementary Material we also show the results when these outcomes are analysed as ordinal and find substantively identical results.

We normalised key variables to the range 0–1 (all variables in Table 1). For all analyses we also conducted sensitivity analyses excluding the two countries with the largest participant numbers: Kazakhstan and Great Britain. Since these two countries make up a majority of our sample excluding them checks if our results also hold in the remaining, reduced, sample.

### Data Sharing

Data and code are publicly available on OSF (https://osf.io/aykgh/, DOI 10.17605/OSF.IO/AYKGH). No identifiers have been collected for this project and hence responses pose no threat to anonymity.

### Ethics

Ethics for this study was received from the ethics committee at London School of Hygiene and Tropical Medicine. Consent to participate is given by submitting the questionnaire as stated in the questionnaire itself (see Supplementary Material).

## Results

The survey received 1,315 responses during the study-period. We excluded 82 observations: 79 because they did not provide location information precluding possible inclusion and three because of suspected duplicate submission. This left a final sample of 1,233 from 77 countries and very few missing values (<1%). The final country list and corresponding number of responses can be found in Supplementary Tables S1, S2. Two countries contribute the majority of responses: Kazakhstan (538 responses) and Great Britain (122 responses). Other countries that contributed substantially include: China (56), Malta (55), Canada (54) and Ukraine (33). The remaining 71 countries have between 27 and 1 respondents (50 have fewer than 5 responses). As a substantial number of countries had few responses, we did not calculate country level estimates.

### Description of Social Expectations, Rewards, Punishment, and Personal Beliefs

We find high levels of social expectations, rewards and punishment, and personal normative beliefs (Supplementary Tables S2, S3, and Supplementary Figure S1). The vast majority of respondents agree that they should always rub/wash their hands before touching a patient (89.9%). Respondents on average reported that approximately 9/10 health workers in their area thought they should always wash/rub their hands before touching a patient (normative expectations). Whilst, when asked how many of their colleagues actually do it, the mean was approximately 7/10 of other health workers in their area (empirical expectations). A majority of respondents also reported that it is always appropriate for a supervisor to challenge someone who did not wash or rub hands before touching a patient (68.0%) and only a small minority believed that this is never appropriate (4.6%). Finally, most respondents had experienced support or promotion of hand hygiene multiple times in the past week (72.5%) while a minority report that this has never happened to them in the past week (14.5%).

There is, however, substantial variation across social expectations. Normative expectations are the highest (0.87, 95% CI = [0.80; 0.94]), followed by empirical expectations (0.75, 95% CI = [0.61; 0.88]), empirical expectations concerning senior staff (0.76, 95% CI = [0.61; 0.91]) and empirical expectations during busy periods (0.71, 95% CI = [0.56; 0.87]). Punishment (0.82, 95% CI = [0.78; 0.85]) and reward (0.79, 95% CI = [0.74; 0.84]) were approximately equal. The relative ordering remains the same without Kazakhstan and Great Britain although the absolute values change somewhat (Supplementary Table S2, Supplementary Figures S2, S3).

In the Supplementary Material, we compare our sample to the relevant population of healthcare workers and find that doctors and females are somewhat overrepresented in our sample. These differences between our sample composition and population composition could bias our estimates. However, in the next section we study the association between these factors and social expectations, rewards punishment, and personal beliefs, allowing us to anticipate potential bias.

The intraclass correlation coefficients range from 0.05 to 0.26 (ICC_PNB_ = 0.16, ICC_NE_ = 0.19, ICC_EE_ = 0.22, ICC_EE busy_ = 0.26, ICC_EE senior_ = 0.23, ICC_Punish_ = 0.05, ICC_Reward_ = 0.10) implying that most variation is between individuals and not countries.

### Assessing the Factors Associated With Social Expectations, Reward, Punishment, and Personal Normative Beliefs

**FIGURE 1:**
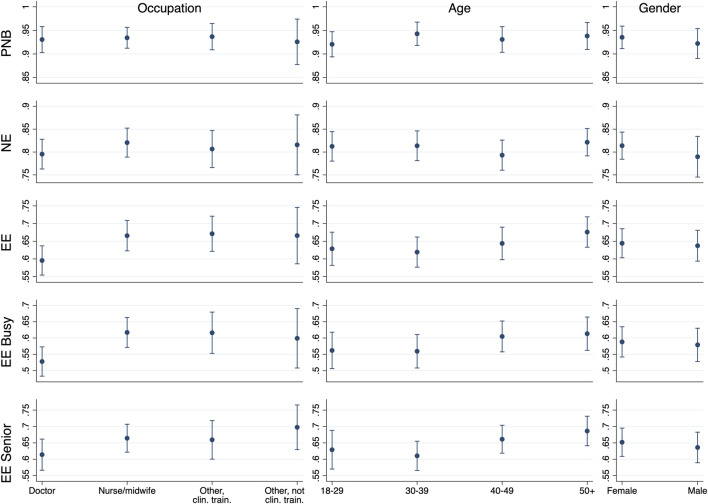
Social expectations and personal normative beliefs according to occupation, age, and gender (77 countries, 2020). Notes: y-axes indicate predicted margins from multilevel models. Error bars represent 95% CIs calculated using multilevel models with cluster robust standard errors at the country level. PNB, personal normative beliefs; NE, normative expectations; EE, empirical expectations; EE busy, empirical expectations when the ward is busy; EE senior, empirical expectations of senior healthcare workers.

We test whether occupation, age, and gender are associated with social expectations and personal normative beliefs (Figure 1, Supplementary Tables S4, S5). For personal normative beliefs and normative expectations there is little evidence for associations. Respondents in the age-range 30–39 may have higher personal normative beliefs than those between 18–29 (*b* = 0.022, *p* = 0.058). And nurses/midwives have higher normative expectations than doctors (*b* = 0.025, *p* = 0.026), while those aged 40–49 may have lower normative expectations than those aged 18–29 (*b* = -0.019, *p* = 0.094).

In contrast, for all three kinds of empirical expectations (EE, EE busy, EE senior), there are clearer associations. Nurses/midwives and other clinically trained workers have higher empirical expectations than doctors (nurses/midwives: *b* = 0.070, *p* < 0.001; other clinically trained: *b* = 0.076, *p* = 0.004), when busy (nurses/midwives: *b* = 0.089, *p* < 0.001; other clinically trained: *b* = 0.088, *p* = 0.007), and about senior staff (nurses/midwives: *b* = 0.050, *p* = 0.005; other clinically trained: *b* = 0.045, *p* = 0.159). Other not clinically trained workers may also have higher empirical expectations than doctors (*b* = 0.070, *p* = 0.068), when busy (*b* = 0.071, *p* = 0.062), and towards senior staff (*b* = 0.083, *p* = 0.004). Age is often, but not always, associated with higher empirical expectations, when busy, and about senior staff. For instance, respondents 50+ have higher empirical expectations than younger age categories (vs. 18–29: *b* = 0.047, *p* = 0.037; vs. 30–39: *b* = 0.057, *p* = 0.009; vs. 40–49: *b* = 0.032, *p* = 0.110). None of the other coefficients approach significance (e.g., gender). We find very similar results when excluding Kazakhstan (Supplementary Figure S4) and Great Britain (Supplementary Figure S5).

**FIGURE 2:**
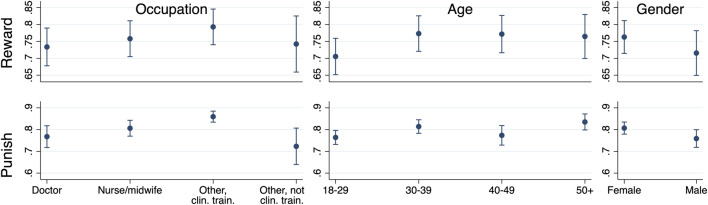
Reward and punishment according to occupation, age, and gender (77 or 76 countries, 2020). Notes: y-axes indicate predicted margins from multilevel models. Error bars represent 95% CIs calculated using multilevel models with cluster robust standard errors at the country level. Reward: experience of reward (77 countries). Punishment: appropriateness of punishment (76 countries).

For rewards we find that other clinically trained workers report slightly higher responses (*b* = 0.059, *p* = 0.032) than doctors and all older age-groups (from 30–39 onwards) report higher responses than the youngest age-group (Figure 2, Supplementary Table S4). There is a weak indication that men report lower reward than women (*b* = −0.047, *p* = 0.093). For punishment, other clinically trained workers report more punishment relative to all other occupational groups (e.g., vs. doctors: *b* = 0.092, *p* < 0.001), older age groups report similar or higher levels of punishment than the youngest age group (30–39 vs. 18–29: *b* = 0.050, *p* = 0.007; 40–49 vs. 18–29: *b* = 0.010, *p* = 0.700; 50+ vs. 18–29: *b* = 0.071, *p* < 0.001), and men report less punishment than women (*b* = −0.048, *p* = 0.025) (Figure 2, Supplementary Table S4). None of the other coefficients approach significance. We find very similar results when excluding Kazakhstan (Supplementary Figure S6) and Great Britain (Supplementary Figure S7).

We next use empirical and normative expectations as the outcomes and the other normative system components as predictors and control for age, gender, occupation, and GDP per capita in the full model (the results below are from the full model; the reduced models also show the same results). Empirical expectations (Supplementary Table S6) are positively associated with personal normative beliefs (*b* = 0.088, *p* = 0.009), normative expectations (*b* = 0.665, *p* < 0.001), and borderline with punishment (*b* = 0.029, *p* = 0.087). Normative expectations (Supplementary Table S7) are meanwhile associated with personal normative beliefs (*b* = 0.202, *p* < 0.001), empirical expectations (*b* = 0.451, *p* < 0.001), and reward (*b* = 0.021, *p* = 0.053). These associations are generally robust to the exclusion of Kazakhstan and Great Britain; the sole exception is that punishment is no longer weakly associated with empirical expectations (Supplementary Tables S8–S11).

### Assessing if Social Expectations Changed During COVID-19

**FIGURE 3:**
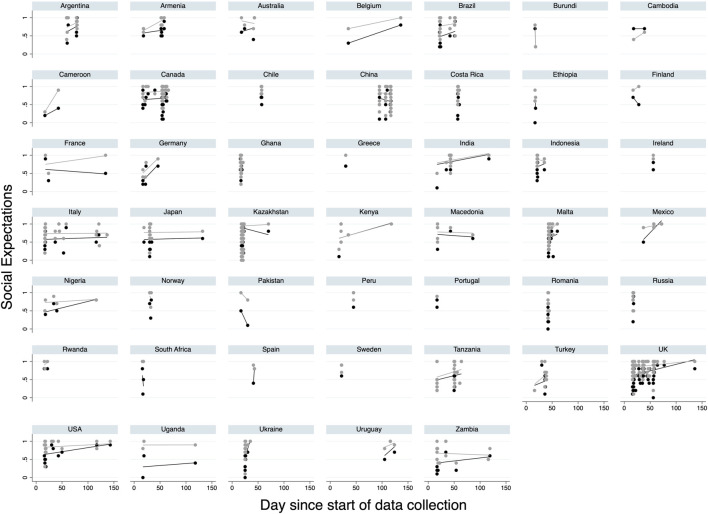
Association between social expectations and day (47 countries, 2020). Notes: y-axes indicate social expectations. Day represents day since first observation (0) in dataset. Black dots represent empirical expectations and grey dots represent normative expectations. Lines indicate OLS fitted bivariate regressions. Only countries with multiple data points included in figure.

Finally, we tested the associations between social expectations and date and find that in a majority of countries expectations either go up with time or stay the same (Figure 3). Formally, empirical expectations are positively associated with day across all models (reduced model: *b* = 0.002, *p* < 0.001; full model: *b* = 0.001, *p* = 0.008; Supplementary Table S12). Normative expectations are likewise positively associated with date across all models (reduced model: *b* = 0.001, *p* < 0.001; full model: *b* = 0.0004, *p* = 0.034; Supplementary Table S13). Both associations are robust to the exclusion of Kazakhstan (Supplementary Tables S14, S15) and the United Kingdom (Supplementary Tables S16, S17). We ran additional models that control for population size, governmental policy stringency, include random effects for date, and change the standard error estimation approach (Supplementary Tables S12, S13) as well using COVID-19 cases and cases/million population (Supplementary Tables S18, S19) and find the same results.

In sum, we find:

• High reported levels of social expectations, personal normative beliefs, punishment, and reward, with normative expectations higher than empirical expectations. Punishment and reward were approximately equal.
• Occupation and age are reliably associated with empirical expectations, somewhat with normative expectations, and little with personal normative beliefs. As anticipated (10, 12), personal empirical beliefs, empirical expectations, and normative expectations are all inter-related.
• Empirical and normative expectations are positively associated with date in the early phases of the COVID-19 pandemic accounting for multiple individual and macro controls.

## Discussion

Using a cross-sectional non-probability online survey, we found that personal normative beliefs and normative expectations are higher than empirical expectations suggesting that practitioners are pessimistic of their colleagues’ values translating into behaviour. This may reflect real hand hygiene compliance levels, which are known to be less than ideal (13). Empirical expectations during busy shifts are also lower than overall empirical expectations which reflect the true adherence of behaviours at these times (14). Interestingly, most variation (ICC) is found between individuals rather than between countries, which follows the pattern of hand hygiene variation in behaviour adherence found for individuals within hospitals in other studies (23). Overall, this consistency between levels and variation in social norms components and observed adherence from other studies suggest our results are credible.

Concerning the demographic factors associated with social norms, occupation is important across the board, except for personal normative beliefs, with doctors having the lowest outcomes. Such lower expectations match the lower hand hygiene that has been reported for doctors (13, 14). Generally, but not always, older respondents have higher outcomes for empirical expectations, reward, and punishment. It is unclear why this is the case. Speculatively, healthcare workers may learn and internalise these norms on the job more so than during their formal education. Potentially, because they are more exposed by hospital-based campaigns such as those promoted by the WHO for the past decade (24).

We also find that empirical expectations are predicted by personal normative beliefs and normative expectations. And normative expectations are predicted by personal normative beliefs and empirical expectations. Consistent with (10, 12), these factors are interconnected and suggests that the framework is applicable to healthcare contexts. Curiously, we find some evidence that punishment appropriateness predicts empirical expectations but not normative expectations, while reward experience predicts normative expectations but not empirical expectations. If robust, this implies that punishment primarily supports behaviour—more specifically people’s expectations about behaviour—but not its normative backing while reward primarily supports the normativity of behaviour but not necessarily expectations about behaviour. This line of reasoning would be consistent with literature that considers punishment as an incentive that can have both positive and negative consequences for prosocial actions (25, 26).

Finally, after accounting for candidate confounders, our results suggest that social expectations increased with the time during the early phase of the COVID-19 pandemic. While this suggests the pandemic increased social expectations, we are unable to identify the specific pathways (since time passing is a proxy for the combination of factors) which we leave to future research.

The main limitation of this study is, in its nature, a non-representative survey of health workers, with an over-representation from female health workers. It is likely to have attracted highly interested and motivated individuals and hence our results should be interpreted with this lens. Specifically, comparing our sample to the population we found an overrepresentation of doctors and female healthcare workers. The latter are unlikely to substantially affect our estimates of social expectations, personal normative beliefs, rewards, and punishment since there are few and small differences in these across genders. While the former may reduce our estimates as doctors were generally found to have lower social expectations than other healthcare workers. Moreover, since the study is observational it is possible that different groups of healthcare workers were responding to our survey at different times introducing selection bias. However, two sets of results suggest that even though our results are not generated from a representative sample, they may still provide generalisable evidence: 1) demographics patterns e.g., by occupation are consistent with the behavioural data from several studies (13, 14); 2) the results of the ICC suggest that most variation lies within rather than between countries. We had a highly variable representation by country and world region, but our sensitivity analyses where we excluded first the UK and then Kazakhstan suggest that all our key results hold and are robust for this self-selected sample of individuals. Respondents from Kazakhstan score higher across all social norms components for absolute values, perhaps reflecting a strong hierarchical system in the healthcare environment in the country or directions from supervisors (although our results hold when excluding respondents from Kazakhstan). Finally, we had limited individual-level confounders when running our models but the aim of the paper was to report on exploratory analysis of social norms on hand hygiene among healthcare workers without attempting to answer causality and without overburdening this key occupation with a long questionnaire during a global crisis.

With these limitations in mind, our findings from 1,233 responses from 77 countries suggest that hand hygiene social norms are strong among health workers and their strength increased during the early stages of COVID-19 pandemic. Patterns of variation, including by demographic variables, are consistent across countries and appear to reflect behavioural data available from other studies. With a striking need to improve hand hygiene among healthcare workers worldwide to avoid healthcare associated infections (27), our findings have a key implication: hand hygiene social norms can change and reflect behavioural patterns; hence norm-targeting interventions should be better embedded in hospital interventions aimed at improving hand hygiene and the wider infection prevention spectrum of behaviour as they have a strong potential to leverage sustained behaviour change. Indeed, when social norms are established, they work *via* a cycle of expectations, behaviour and punishment/rewards that provides a means to sustain behaviour.
