# Supplementary material for: Hand Hygiene Social Norms Among Healthcare Workers During Early COVID-19: Results of a Global Survey
Source: Int J Public Health. 2022 Nov 24;67:1604981. doi: 10.3389/ijph.2022.1604981 (PMC9729248; doi:10.3389/ijph.2022.1604981)
Supplement: Supplementary file 1 [file DataSheet1.docx]

Supplementary Materials for

**Hand Hygiene Social Norms among Healthcare Workers During Early COVID-19: Results of a Global Survey**

Survey (English Language Version) 3

Sample and Population Comparison 6

Networks Approached 7

Supplementary Tables 8

**Table S1**. Countries and observations (77 countries, 2020). 8

**Table S2**. Descriptive characteristics of sample (77, 76, or 75 countries, 2020). 10

**Table S3**. Personal normative beliefs, punishment, and reward (77 or 76 countries, 2020). 11

**Table S4**. Multilevel model outputs predicting social expectations, reward, punishment, and personal beliefs (77 or 76 countries, 2020). 12

**Table S5**. Multilevel ordered logit regression predicting personal normative beliefs, reward, and punishment (77 countries, 2020). 13

**Table S6**. Multilevel model outputs of the normative system variables predicting empirical expectations (76 or 74 countries, 2020). 14

**Table S7**. Multilevel model outputs of the normative system variables predicting normative expectations (76 or 75 countries, 2020). 15

**Table S8**. Multilevel model outputs of the normative system variables predicting empirical expectations. Excluding Kazakhstan (75 or 73 countries, 2020). 16

**Table S9**. Multilevel model outputs of the normative system variables predicting empirical expectations. Excluding Great Britain (75 or 73 countries, 2020). 17

**Table S10**. Multilevel model outputs of the normative system variables predicting normative expectations. Excluding Kazakhstan (75 or 73 countries, 2020). 18

**Table S11**. Multilevel model outputs of the normative system variables predicting normative expectations. Excluding Great Britain (75 or 73 countries, 2020). 19

**Table S12**. Empirical expectations and date. Multilevel model outputs including full sample (77, 74, or 71 countries, 2020). 20

**Table S13**. Normative expectations and day. Multilevel model including full sample (77, 74, or 71 countries, 2020). 22

**Table S14**. Empirical expectations and date. Multilevel model outputs excluding Kazakhstan (76 or 73 countries, 2020). 24

**Table S15**. Normative expectations and day. Multilevel model excluding Kazakhstan (76 or 73 countries, 2020). 25

**Table S16**. Empirical expectations and date. Multilevel model outputs excluding Great Britain (76 or 73 countries, 2020). 26

**Table S17**. Normative expectations and day. Multilevel model excluding Great Britain (76 or 73 countries, 2020). 27

**Table S18**. Empirical expectations and COVID-19 cases. Multilevel model outputs including full sample (77 countries, 2020). 28

**Table S19**. Normative expectations and COVID-19 cases. Multilevel model outputs including full sample (77 countries, 2020). 29

Supplementary Figures 30

**Figure S1**. Full sample: histograms of personal normative beliefs, social expectations (NE, EE, EE busy, EE senior), punishment, and reward (77 or 76 countries, 2020). 30

**Figure S2**. Sample excluding Kazakhstan: histograms of personal normative beliefs, social expectations (NE, EE, EE busy, EE senior), punishment, and reward (76 or 75 countries, 2020). 31

**Figure S3**. Sample excluding Great Britain: histograms of personal normative beliefs, social expectations (NE, EE, EE busy, EE senior), punishment, and reward (76 or 75 countries, 2020). 32

**Figure S4**. Social expectations and personal normative beliefs according to occupation, age, and gender. Kazakhstan excluded (76 countries, 2020). 33

**Figure S5**. Social expectations and personal normative beliefs according to occupation, age, and gender. Great Britain excluded (76 countries, 2020). 34

**Figure S6**. Reward and punishment according to occupation, age, and gender. Kazakhstan excluded (76 or 75 countries, 2020). 35

**Figure S7**. Reward and punishment according to occupation, age, and gender. Great Britain excluded (76 or 75 countries, 2020). 36

References 37

# Survey (English Language Version)


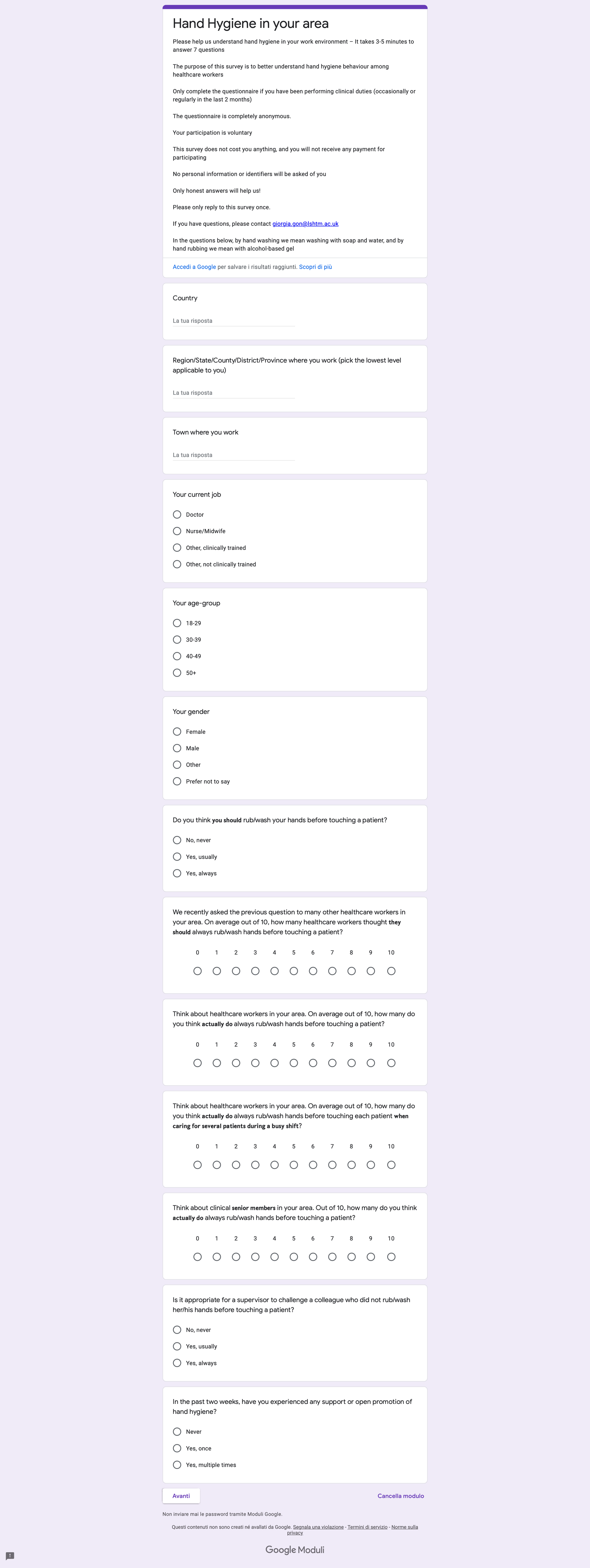


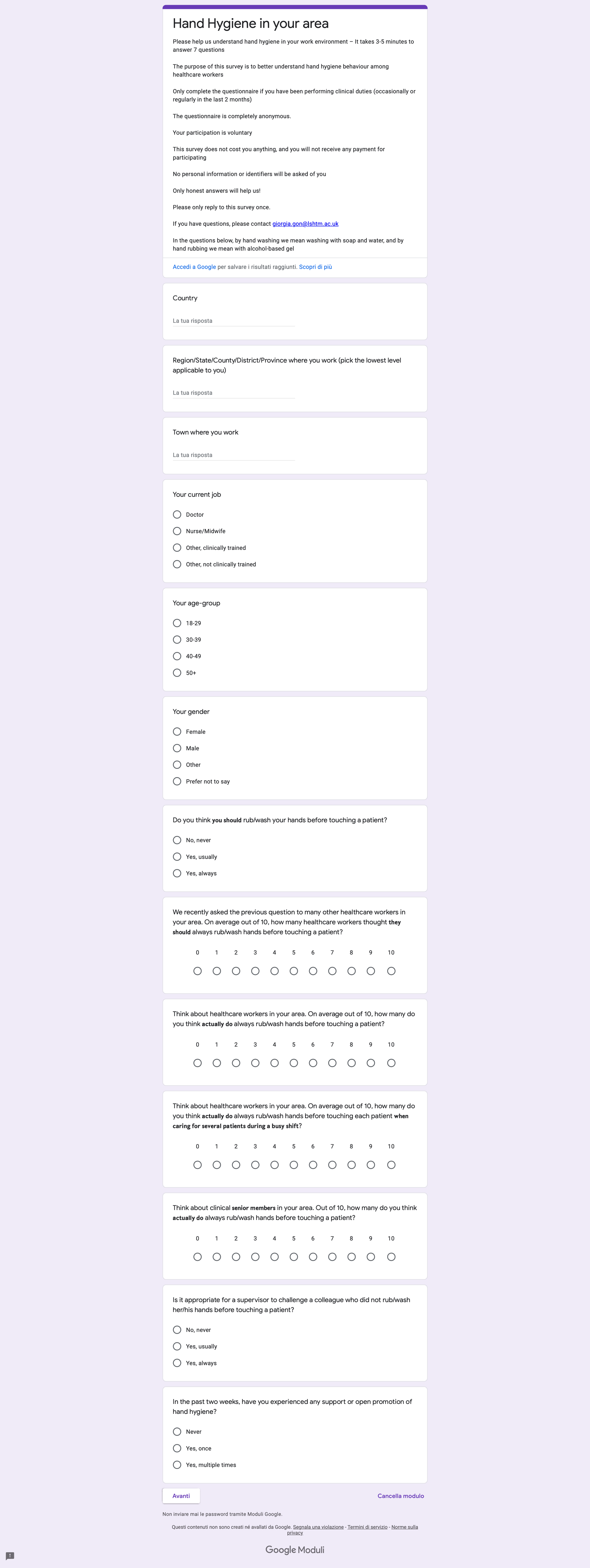


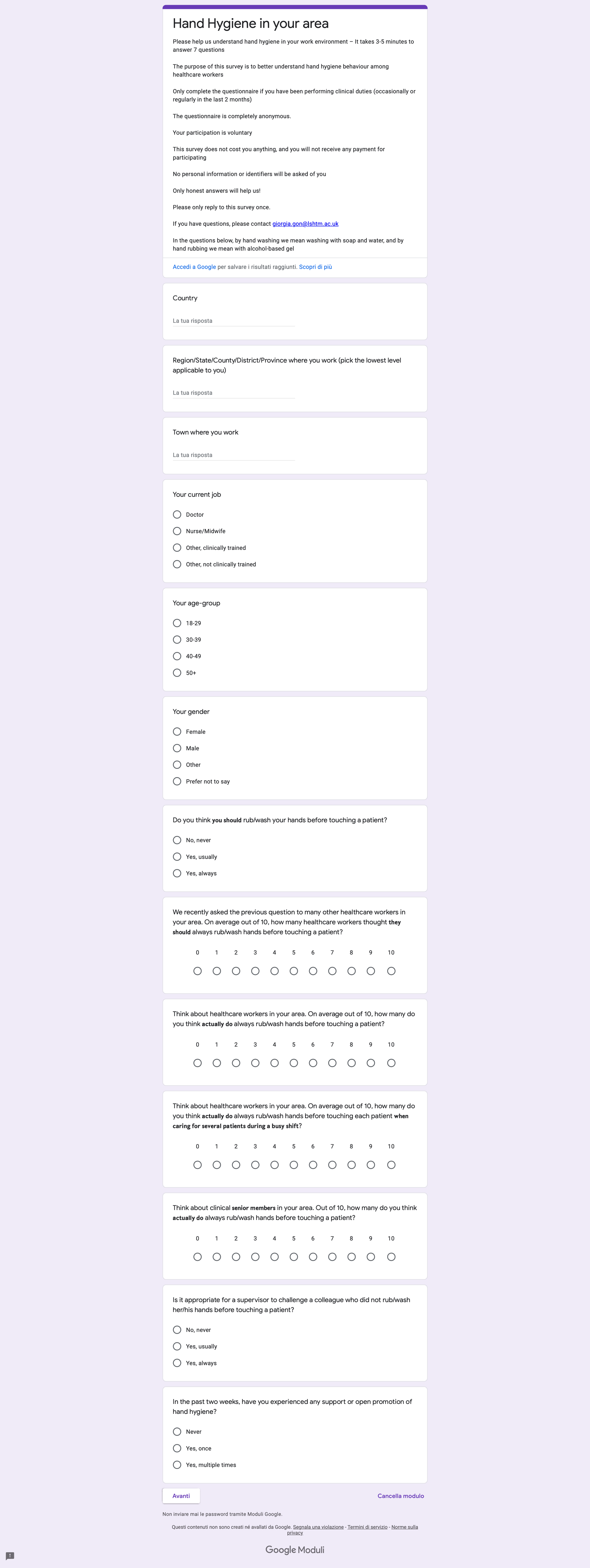


# Sample and Population Comparison

To compare our sample with the target population, we retrieved the latest available population data from the WHO Global Health Workforce statistics database on healthcare workers. Specifically, we made three comparisons:

1. The ratio of nurses (total number) to doctors (total number) in the population to the ratio of nurses to doctors in our sample. This comparison was done for all 77 countries in our sample as all sample countries are present in the WHO data.
2. The ratio of female to male doctors in the population to the ratio of female to male doctors in our sample. We conducted this comparison for a subset of 48 countries that were contained in both the WHO population data and our sample data.
3. The ratio of female to male nurses in the population to the ratio of female to male nurses in our sample. We conducted this for a subset of 26 countries that were both in the WHO population data and our sample data.

For all of these comparisons, we match weight of each country in the target population data to the weight that those countries have in our sample.

We find that we have (i) fewer nurses relative to doctors (sample ratio: 1.43; population ratio: 2.09), (ii) more female doctors relative to male doctors (sample ratio: 1.85; population ratio: 0.98), and (iii) more female nurses relative to male nurses (sample ratio: 9.00; population ratio: 6.60) than would be expected from a representative sample of our countries.

# Networks Approached

1. LSHTM Maternal Health Group
2. LSHTM students
3. WhatsApp group: Health Psychology and COVID-19 (researcher’s group)
4. WHO Euro Infection Prevention and Control Country Network
5. Africa Centre for Disease Control network
6. FIGO – International Federation of Gynecology and Obstetrics
7. Embedded in the second round of a global survey on maternal health services during COVID-19 (1)
8. Hertfordshire Community NHS Trust
9. FNOPO – Italy
10. American Nurses Association Enterprise Customer Care Center
11. Canadian Nurses Association
12. Twitter:
13. UK Healthcare Infection Society
14. Canadian Midwifes
15. Global Health Networks
16. Hannover Medical School
17. Individual colleagues who circulated it to their contacts and lists

# Supplementary Tables

**Table S1**. Countries and observations (77 countries, 2020).

| **Country** | **Frequency** | **Percent** |
| --- | --- | --- |
| Kazakhstan | 538 | 43.63 |
| United Kingdom | 122 | 9.89 |
| China | 56 | 4.54 |
| Malta | 55 | 4.46 |
| Canada | 54 | 4.38 |
| Ukraine | 33 | 2.68 |
| Italy | 27 | 2.19 |
| Tanzania | 25 | 2.03 |
| Ghana | 23 | 1.87 |
| Japan | 23 | 1.87 |
| India | 21 | 1.70 |
| USA | 21 | 1.70 |
| Brazil | 17 | 1.38 |
| Russian | 17 | 1.38 |
| Indonesia | 13 | 1.05 |
| Zambia | 13 | 1.05 |
| Romania | 12 | 0.97 |
| Turkey | 12 | 0.97 |
| Chile | 11 | 0.89 |
| Costa Rica | 10 | 0.81 |
| Argentina | 9 | 0.73 |
| Armenia | 8 | 0.65 |
| Macedonia | 8 | 0.65 |
| Kenya | 7 | 0.57 |
| Nigeria | 6 | 0.49 |
| Ethiopia | 5 | 0.41 |
| Germany | 5 | 0.41 |
| Australia | 4 | 0.32 |
| Mexico | 4 | 0.32 |
| France | 3 | 0.24 |
| Greece | 3 | 0.24 |
| Norway | 3 | 0.24 |
| Peru | 3 | 0.24 |
| Rwanda | 3 | 0.24 |
| South Africa | 3 | 0.24 |
| Uganda | 3 | 0.24 |
| Uruguay | 3 | 0.24 |
| Belgium | 2 | 0.16 |
| Burundi | 2 | 0.16 |
| Cambodia | 2 | 0.16 |
| Cameroon | 2 | 0.16 |
| Finland | 2 | 0.16 |
| Ireland | 2 | 0.16 |
| Pakistan | 2 | 0.16 |
| Portugal | 2 | 0.16 |
| Spain | 2 | 0.16 |
| Sweden | 2 | 0.16 |
| Albania | 1 | 0.08 |
| Azerbaijan | 1 | 0.08 |
| Bangladesh | 1 | 0.08 |
| Bolivia | 1 | 0.08 |
| Botswana | 1 | 0.08 |
| Colombia | 1 | 0.08 |
| Croatia | 1 | 0.08 |
| Denmark | 1 | 0.08 |
| Gambia | 1 | 0.08 |
| Georgia | 1 | 0.08 |
| Guatemala | 1 | 0.08 |
| Honduras | 1 | 0.08 |
| Ivory Coast | 1 | 0.08 |
| Jamaica | 1 | 0.08 |
| Jordan | 1 | 0.08 |
| Lebanon | 1 | 0.08 |
| Morocco | 1 | 0.08 |
| Mozambique | 1 | 0.08 |
| Netherlands | 1 | 0.08 |
| Philippines | 1 | 0.08 |
| Senegal | 1 | 0.08 |
| Slovenia | 1 | 0.08 |
| Somaliland | 1 | 0.08 |
| South Sudan | 1 | 0.08 |
| Sri Lanka | 1 | 0.08 |
| Syria | 1 | 0.08 |
| Uzbekistan | 1 | 0.08 |
| Vietnam | 1 | 0.08 |
| Yemen | 1 | 0.08 |
| Zimbabwe | 1 | 0.08 |
| **Total** | 1233 | 100 |

**Table S2**. Descriptive characteristics of sample (77, 76, or 75 countries, 2020).

| **Variable** | **Full sample**  (n*_i_*=1,233) | | **Excluding**  **Kazakhstan** (n*_i_*=695) | **Excluding**  **UK**  (n*_i_*=1,111) |
| --- | --- | --- | --- | --- |
|  | n*_i_* (missing) | Mean or % (SD) | Mean  (SD) | Mean (SD) |
| Personal normative beliefs (PNB) | 1,232 (1) | 0.95 (0.16) | 0.93 (0.18) | 0.95 (0.16) |
| Normative expectations (NE) | 1,229 (4) | 0.87 (0.20) | 0.81 (0.21) | 0.87 (0.20) |
| Empirical expectations (EE) | 1,229 (4) | 0.75 (0.26) | 0.63 (0.25) | 0.75 (0.26) |
| Empirical expectations: for senior staff (EE senior) | 1,227 (6) | 0.76 (0.28) | 0.63 (0.28) | 0.77 (0.27) |
| Empirical expectations: when busy (EE busy) | 1,227 (6) | 0.71 (0.29) | 0.58 (0.27) | 0.72 (0.29) |
| Punish | 1,230 (3) | 0.82 (0.28) | 0.79 (0.29) | 0.81 (0.29) |
| Reward | 1,227 (6) | 0.79 (0.37) | 0.75 (0.38) | 0.79 (0.36) |
| **Gender** | 1,232 (1) |  |  |  |
| Male | 211 | 17.11% | 26.62% | 17.64% |
| Female | 1014 | 82.24% | 72.81% | 81.73% |
| Prefer not say | 7 | 0.57% | 0.43% | 0.54% |
| **Age-group** | 1,232 (1) |  |  |  |
| 18-29 | 298 | 24.17% | 23.60% | 23.13% |
| 30-39 | 277 | 22.47% | 29.50% | 23.31% |
| 40-49 | 328 | 26.60% | 24.89% | 27.18% |
| 50+ | 329 | 26.68% | 22.01% | 26.28% |
| **Current job** | 1,230 (3) |  |  |  |
| Doctor | 406 | 32.93% | 43.45% | 33.21% |
| Nurse/midwife | 581 | 47.12% | 35.54% | 48.06% |
| Other clinically trained | 178 | 14.44% | 15.54% | 13.05% |
| Other not clinically trained | 65 | 5.27% | 5.47% | 5.40% |

**Table S3**. Personal normative beliefs, punishment, and reward (77 or 76 countries, 2020).

|  | **Personal normative beliefs** | **Punishment** |  | **Reward** |
| --- | --- | --- | --- | --- |
| **No, never** | 0.3% (4) | 4.6% (56) | **No, never** | 14.5% (178) |
| **Yes, usually** | 9.7% (120) | 27.5% (338) | **Yes, once** | 13.0% (160) |
| **Yes, always** | 89.9% (1,108) | 68.0% (836) | **Yes, multiple times** | 72.5% (889) |

Notes: Column percentages may not add up to 100 because of rounding.

The vast majority of respondents agree that they should always rub/wash their hands before touching a patient (89.9%) and almost nobody reported they should never do so (0.3%). A majority of respondents also reported that it is always appropriate for a supervisor to challenge someone who did not wash or rub hands before touching a patient (68.0%) and only a small minority believed that this is never appropriate (4.6%). Finally, most respondents had experienced support or promotion of hand hygiene multiple times in the past week (72.5%) while a minority report that this has never happened to them in the past week (14.5%).

**Table S4**. Multilevel model outputs predicting social expectations, reward, punishment, and personal beliefs (77 or 76 countries, 2020).

|  | PNB | NE | EE | EE Busy | EE Senior | Reward | Punish |
| --- | --- | --- | --- | --- | --- | --- | --- |
| *Doctor (Ref. cat.)* | - | - | - | - | - | - | - |
|  |  |  |  |  |  |  |  |
| Nurse/Midwife | 0.004 | 0.025^*^ | 0.070^***^ | 0.089^***^ | 0.050^**^ | 0.024 | 0.039 |
|  | (0.010) | (0.011) | (0.016) | (0.016) | (0.018) | (0.022) | (0.033) |
| Other, clinically trained | 0.006 | 0.011 | 0.076^**^ | 0.088^**^ | 0.045 | 0.059^*^ | 0.092^***^ |
|  | (0.015) | (0.024) | (0.026) | (0.033) | (0.032) | (0.028) | (0.022) |
| Other, not clinically trained | -0.005 | 0.020 | 0.070^+^ | 0.071^+^ | 0.083^**^ | 0.009 | -0.045 |
|  | (0.024) | (0.026) | (0.038) | (0.038) | (0.029) | (0.037) | (0.055) |
| *Female (Ref. cat.)* | - | - | - | - | - | - | - |
|  |  |  |  |  |  |  |  |
| Male | -0.013 | -0.024 | -0.007 | -0.009 | -0.016 | -0.047^+^ | -0.048^*^ |
|  | (0.016) | (0.021) | (0.020) | (0.026) | (0.023) | (0.028) | (0.021) |
| Prefer not to say | -0.023 | 0.032 | 0.039 | -0.014 | -0.090 | -0.129 | -0.347^**^ |
|  | (0.056) | (0.051) | (0.027) | (0.078) | (0.078) | (0.090) | (0.108) |
| *18-29 (Ref. cat.)* | - | - | - | - | - | - | - |
|  |  |  |  |  |  |  |  |
| 30-39 | 0.022^+^ | 0.001 | -0.009 | -0.003 | -0.019 | 0.068^**^ | 0.050^**^ |
|  | (0.012) | (0.011) | (0.015) | (0.019) | (0.023) | (0.023) | (0.018) |
| 40-49 | 0.010 | -0.019^+^ | 0.015 | 0.043^**^ | 0.032 | 0.066^**^ | 0.010 |
|  | (0.013) | (0.011) | (0.013) | (0.014) | (0.020) | (0.022) | (0.025) |
| 50+ | 0.018 | 0.009 | 0.047^*^ | 0.051 | 0.057^+^ | 0.059^*^ | 0.071^***^ |
|  | (0.013) | (0.011) | (0.023) | (0.032) | (0.031) | (0.023) | (0.016) |
| Constant | 0.920^***^ | 0.802^***^ | 0.582^***^ | 0.505^***^ | 0.598^***^ | 0.694^***^ | 0.745^***^ |
|  | (0.016) | (0.020) | (0.028) | (0.033) | (0.039) | (0.031) | (0.024) |
| SD(Constant) | 0.064^***^ | 0.086^***^ | 0.111^***^ | 0.130^***^ | 0.120^***^ | 0.122^***^ | 0.045^***^ |
|  | (0.013) | (0.021) | (0.021) | (0.024) | (0.026) | (0.038) | (0.015) |
| SD(Residual) | 0.148^***^ | 0.181^***^ | 0.214^***^ | 0.224^***^ | 0.222^***^ | 0.351^***^ | 0.275^***^ |
|  | (0.016) | (0.013) | (0.012) | (0.014) | (0.023) | (0.011) | (0.005) |
| Observations (individuals) | 1227 | 1224 | 1224 | 1222 | 1222 | 1222 | 1225 |
| Observations (groups) | 77 | 77 | 77 | 77 | 77 | 77 | 76 |

Standard errors in parentheses

^+^ *p* < 0.10, ^*^ *p* < 0.05, ^**^ *p* < 0.01, ^***^ *p* < 0.001

**Table S5**. Multilevel ordered logit regression predicting personal normative beliefs, reward, and punishment (77 countries, 2020).

|  | PNB | Reward | Punish |
| --- | --- | --- | --- |
| *Doctor (Ref. cat.)* | - | - | - |
|  |  |  |  |
| Nurse/Midwife | 0.147 | 0.164 | 0.336 |
|  | (0.226) | (0.155) | (0.242) |
| Other, clinically trained | 0.230 | 0.339^+^ | 0.755^***^ |
|  | (0.295) | (0.186) | (0.171) |
| Other, not clinically trained | 0.051 | 0.025 | -0.213 |
|  | (0.466) | (0.207) | (0.372) |
| *Female (Ref. cat.)* | - | - | - |
|  |  |  |  |
| Male | -0.285 | -0.303^+^ | -0.372^*^ |
|  | (0.264) | (0.159) | (0.153) |
| Prefer not to say | -0.560 | -1.037^*^ | -2.221^***^ |
|  | (0.911) | (0.445) | (0.640) |
| *18-29 (Ref. cat.)* | - | - | - |
|  |  |  |  |
| 30-39 | 0.446 | 0.403^**^ | 0.361^*^ |
|  | (0.278) | (0.139) | (0.143) |
| 40-49 | 0.218 | 0.474^**^ | 0.128 |
|  | (0.313) | (0.171) | (0.186) |
| 50+ | 0.488 | 0.392^**^ | 0.652^***^ |
|  | (0.327) | (0.139) | (0.151) |
| Cut 1 | -5.575 | -1.300 | -2.571 |
|  | (0.462) | (0.181) | (0.159) |
| Cut 2 | -1.885 | -0.435 | -0.178 |
|  | (0.280) | (0.173) | (0.178) |
| Var(Constant) | 0.877 | 0.395 | 0.100 |
|  | (0.311) | (0.209) | (0.064) |
| Observations (individuals) | 1227 | 1222 | 1225 |
| Observations (groups) | 77 | 77 | 77 |

Standard errors in parentheses

^+^ *p* < 0.10, ^*^ *p* < 0.05, ^**^ *p* < 0.01, ^***^ *p* < 0.001

**Table S6**. Multilevel model outputs of the normative system variables predicting empirical expectations (76 or 74 countries, 2020).

|  | Model 1 | Model 2 |
| --- | --- | --- |
| Personal normative beliefs | 0.083^*^ | 0.088^**^ |
|  | (0.033) | (0.034) |
| Normative expectations | 0.667^***^ | 0.665^***^ |
|  | (0.035) | (0.035) |
| Reward | 0.023 | 0.020 |
|  | (0.018) | (0.016) |
| Punish | 0.035^+^ | 0.029^+^ |
|  | (0.018) | (0.017) |
| *Female (Ref. cat.)* |  | - |
|  |  |  |
| Male |  | 0.012 |
|  |  | (0.016) |
| Prefer not to say |  | 0.022 |
|  |  | (0.018) |
| *Doctor (Ref. cat.)* |  | - |
|  |  |  |
| Nurse/Midwife |  | 0.053^***^ |
|  |  | (0.011) |
| Other, clinically trained |  | 0.063^***^ |
|  |  | (0.019) |
| Other, not clinically trained |  | 0.060^+^ |
|  |  | (0.032) |
| *18-29 (Ref. cat.)* |  | - |
|  |  |  |
| 30-39 |  | -0.021^+^ |
|  |  | (0.012) |
| 40-49 |  | 0.027^**^ |
|  |  | (0.009) |
| 50+ |  | 0.038^*^ |
|  |  | (0.018) |
| GDP per capita |  | -0.000 |
|  |  | (0.000) |
| Constant | -0.028 | -0.053 |
|  | (0.048) | (0.053) |
| SD(Constant) | 0.084^***^ | 0.078^***^ |
|  | (0.020) | (0.019) |
| SD(Residual) | 0.176^***^ | 0.173^***^ |
|  | (0.006) | (0.006) |
| Observations (individuals) | 1219 | 1212 |
| Observations (groups) | 76 | 74 |

Standard errors in parentheses

^+^ *p* < 0.10, ^*^ *p* < 0.05, ^**^ *p* < 0.01, ^***^ *p* < 0.001

**Table S7**. Multilevel model outputs of the normative system variables predicting normative expectations (76 or 75 countries, 2020).

|  | Model 1 | Model 2 |
| --- | --- | --- |
| Personal normative beliefs | 0.211^***^ | 0.202^***^ |
|  | (0.030) | (0.030) |
| Empirical expectations | 0.443^***^ | 0.451^***^ |
|  | (0.041) | (0.041) |
| Reward | 0.021^+^ | 0.021^+^ |
|  | (0.011) | (0.011) |
| Punish | 0.002 | 0.003 |
|  | (0.017) | (0.016) |
| *Female (Ref. cat.)* |  | - |
|  |  |  |
| Male |  | -0.019 |
|  |  | (0.016) |
| Prefer not to say |  | 0.028 |
|  |  | (0.027) |
| *Doctor (Ref. cat.)* |  | - |
|  |  |  |
| Nurse/Midwife |  | -0.012 |
|  |  | (0.008) |
| Other, clinically trained |  | -0.021^*^ |
|  |  | (0.010) |
| Other, not clinically trained |  | -0.017 |
|  |  | (0.022) |
| *18-29 (Ref. cat.)* |  | - |
|  |  |  |
| 30-39 |  | 0.007 |
|  |  | (0.008) |
| 40-49 |  | -0.026^**^ |
|  |  | (0.008) |
| 50+ |  | -0.018^+^ |
|  |  | (0.010) |
| GDP per capita |  | 0.000^*^ |
|  |  | (0.000) |
| Constant | 0.309^***^ | 0.308^***^ |
|  | (0.027) | (0.027) |
| SD(Constant) | 0.053^***^ | 0.039^***^ |
|  | (0.019) | (0.022) |
| SD(Residual) | 0.144^***^ | 0.144^***^ |
|  | (0.007) | (0.008) |
| Observations (individuals) | 1219 | 1212 |
| Observations (groups) | 76 | 75 |

Standard errors in parentheses

^+^ *p* < 0.10, ^*^ *p* < 0.05, ^**^ *p* < 0.01, ^***^ *p* < 0.001

**Table S8**. Multilevel model outputs of the normative system variables predicting empirical expectations. Excluding Kazakhstan (75 or 73 countries, 2020).

|  | Model 1 | Model 2 |
| --- | --- | --- |
| Personal normative beliefs | 0.096^*^ | 0.099^*^ |
|  | (0.040) | (0.042) |
| Normative expectations | 0.706^***^ | 0.704^***^ |
|  | (0.035) | (0.034) |
| Reward | 0.003 | 0.004 |
|  | (0.026) | (0.023) |
| Punish | 0.029 | 0.020 |
|  | (0.033) | (0.029) |
| *Female (Ref. cat.)* |  | - |
|  |  |  |
| Male |  | 0.023 |
|  |  | (0.016) |
| Prefer not to say |  | 0.055^+^ |
|  |  | (0.032) |
| *Doctor (Ref. cat.)* |  | - |
|  |  |  |
| Nurse/Midwife |  | 0.051^**^ |
|  |  | (0.019) |
| Other, clinically trained |  | 0.058^+^ |
|  |  | (0.029) |
| Other, not clinically trained |  | 0.070 |
|  |  | (0.051) |
| *18-29 (Ref. cat.)* |  | - |
|  |  |  |
| 30-39 |  | -0.019 |
|  |  | (0.018) |
| 40-49 |  | 0.021 |
|  |  | (0.018) |
| 50+ |  | 0.065^**^ |
|  |  | (0.020) |
| GDP per capita |  | -0.000 |
|  |  | (0.000) |
| Constant | -0.057 | -0.083 |
|  | (0.055) | (0.058) |
| SD(Constant) | 0.067^***^ | 0.063^***^ |
|  | (0.024) | (0.023) |
| SD(Residual) | 0.185^***^ | 0.182^***^ |
|  | (0.005) | (0.005) |
| Observations (individuals) | 688 | 685 |
| Observations (groups) | 75 | 73 |

Standard errors in parentheses

^+^ *p* < 0.10, ^*^ *p* < 0.05, ^**^ *p* < 0.01, ^***^ *p* < 0.001

**Table S9**. Multilevel model outputs of the normative system variables predicting empirical expectations. Excluding Great Britain (75 or 73 countries, 2020).

|  | Model 1 | Model 2 |
| --- | --- | --- |
| Personal normative beliefs | 0.073^*^ | 0.079^*^ |
|  | (0.035) | (0.036) |
| Normative expectations | 0.662^***^ | 0.663^***^ |
|  | (0.038) | (0.038) |
| Reward | 0.014 | 0.013 |
|  | (0.021) | (0.019) |
| Punish | 0.022 | 0.019 |
|  | (0.017) | (0.017) |
| *Female (Ref. cat.)* |  | - |
|  |  |  |
| Male |  | 0.013 |
|  |  | (0.018) |
| Prefer not to say |  | 0.023 |
|  |  | (0.019) |
| *Doctor (Ref. cat.)* |  | - |
|  |  |  |
| Nurse/Midwife |  | 0.048^***^ |
|  |  | (0.011) |
| Other, clinically trained |  | 0.050^**^ |
|  |  | (0.019) |
| Other, not clinically trained |  | 0.036 |
|  |  | (0.024) |
| *18-29 (Ref. cat.)* |  | - |
|  |  |  |
| 30-39 |  | -0.018 |
|  |  | (0.013) |
| 40-49 |  | 0.023^*^ |
|  |  | (0.011) |
| 50+ |  | 0.031^*^ |
|  |  | (0.015) |
| GDP per capita |  | -0.000 |
|  |  | (0.000) |
| Constant | 0.000 | -0.024 |
|  | (0.039) | (0.045) |
| SD(Constant) | 0.088^***^ | 0.082^***^ |
|  | (0.019) | (0.019) |
| SD(Residual) | 0.173^***^ | 0.172^***^ |
|  | (0.005) | (0.005) |
| Observations (individuals) | 1097 | 1090 |
| Observations (groups) | 75 | 73 |

Standard errors in parentheses

^+^ *p* < 0.10, ^*^ *p* < 0.05, ^**^ *p* < 0.01, ^***^ *p* < 0.001

**Table S10**. Multilevel model outputs of the normative system variables predicting normative expectations. Excluding Kazakhstan (75 or 73 countries, 2020).

|  | Model 1 | Model 2 |
| --- | --- | --- |
| Personal normative beliefs | 0.183^***^ | 0.169^***^ |
|  | (0.035) | (0.035) |
| Empirical expectations | 0.486^***^ | 0.499^***^ |
|  | (0.046) | (0.042) |
| Reward | 0.032^*^ | 0.028^+^ |
|  | (0.016) | (0.016) |
| Punish | 0.022 | 0.022 |
|  | (0.022) | (0.022) |
| *Female (Ref. cat.)* |  | - |
|  |  |  |
| Male |  | -0.024 |
|  |  | (0.019) |
| Prefer not to say |  | -0.009 |
|  |  | (0.065) |
| *Doctor (Ref. cat.)* |  | - |
|  |  |  |
| Nurse/Midwife |  | -0.023 |
|  |  | (0.015) |
| Other, clinically trained |  | -0.014 |
|  |  | (0.017) |
| Other, not clinically trained |  | -0.049^+^ |
|  |  | (0.028) |
| *18-29 (Ref. cat.)* |  | - |
|  |  |  |
| 30-39 |  | 0.011 |
|  |  | (0.014) |
| 40-49 |  | -0.017 |
|  |  | (0.014) |
| 50+ |  | -0.027 |
|  |  | (0.019) |
| GDP per capita |  | 0.000^**^ |
|  |  | (0.000) |
| Constant | 0.285^***^ | 0.294^***^ |
|  | (0.031) | (0.027) |
| SD(Constant) | 0.044^***^ | 0.022^***^ |
|  | (0.014) | (0.025) |
| SD(Residual) | 0.155^***^ | 0.155^***^ |
|  | (0.006) | (0.007) |
| Observations (individuals) | 688 | 685 |
| Observations (groups) | 75 | 73 |

Standard errors in parentheses

^+^ *p* < 0.10, ^*^ *p* < 0.05, ^**^ *p* < 0.01, ^***^ *p* < 0.001

**Table S11**. Multilevel model outputs of the normative system variables predicting normative expectations. Excluding Great Britain (75 or 73 countries, 2020).

|  | Model 1 | Model 2 |
| --- | --- | --- |
| Personal normative beliefs | 0.206^***^ | 0.195^***^ |
|  | (0.033) | (0.034) |
| Empirical expectations | 0.457^***^ | 0.462^***^ |
|  | (0.048) | (0.048) |
| Reward | 0.024^+^ | 0.023^+^ |
|  | (0.013) | (0.012) |
| Punish | 0.001 | 0.000 |
|  | (0.018) | (0.017) |
| *Female (Ref. cat.)* |  | - |
|  |  |  |
| Male |  | -0.026 |
|  |  | (0.017) |
| Prefer not to say |  | 0.011 |
|  |  | (0.031) |
| *Doctor (Ref. cat.)* |  | - |
|  |  |  |
| Nurse/Midwife |  | -0.015 |
|  |  | (0.009) |
| Other, clinically trained |  | -0.017 |
|  |  | (0.013) |
| Other, not clinically trained |  | -0.008 |
|  |  | (0.020) |
| *18-29 (Ref. cat.)* |  | - |
|  |  |  |
| 30-39 |  | 0.009 |
|  |  | (0.009) |
| 40-49 |  | -0.028^**^ |
|  |  | (0.009) |
| 50+ |  | -0.014 |
|  |  | (0.009) |
| GDP per capita |  | 0.000^*^ |
|  |  | (0.000) |
| Constant | 0.303^***^ | 0.309^***^ |
|  | (0.032) | (0.031) |
| SD(Constant) | 0.053^***^ | 0.040^***^ |
|  | (0.020) | (0.022) |
| SD(Residual) | 0.145^***^ | 0.145^***^ |
|  | (0.008) | (0.009) |
| Observations (individuals) | 1097 | 1090 |
| Observations (groups) | 75 | 73 |

Standard errors in parentheses

^+^ *p* < 0.10, ^*^ *p* < 0.05, ^**^ *p* < 0.01, ^***^ *p* < 0.001

**Table S12**. Empirical expectations and date. Multilevel model outputs including full sample (77, 74, or 71 countries, 2020).

|  | Model 1 | Model 2 | Model 3 | Model 4 | Model 5 | Model 6 | Model 7 |
| --- | --- | --- | --- | --- | --- | --- | --- |
| Day | 0.002^***^ | 0.001^**^ | 0.001^**^ | 0.001^**^ | 0.001^**^ | 0.001^**^ | 0.001^*^ |
|  | (0.000) | (0.000) | (0.000) | (0.000) | (0.000) | (0.000) | (0.000) |
| *Female (Ref. cat.)* |  | - | - | - | - | - | - |
|  |  |  |  |  |  |  |  |
| Male |  | -0.004 | 0.014 | 0.014 | 0.017 | 0.017 | 0.017 |
|  |  | (0.021) | (0.016) | (0.016) | (0.017) | (0.017) | (0.016) |
| Prefer not to say |  | 0.032 | 0.019 | 0.019 | 0.017 | 0.017 | 0.017 |
|  |  | (0.024) | (0.017) | (0.017) | (0.018) | (0.018) | (0.071) |
| *Doctor (Ref. cat.)* |  | - | - | - | - | - | - |
|  |  |  |  |  |  |  |  |
| Nurse/Midwife |  | 0.063^***^ | 0.049^***^ | 0.049^***^ | 0.045^***^ | 0.045^***^ | 0.045^**^ |
|  |  | (0.015) | (0.011) | (0.011) | (0.012) | (0.012) | (0.014) |
| Other, clinically trained |  | 0.074^**^ | 0.062^***^ | 0.062^***^ | 0.071^***^ | 0.071^***^ | 0.071^***^ |
|  |  | (0.025) | (0.018) | (0.018) | (0.015) | (0.015) | (0.017) |
| Other, not clinically trained |  | 0.064^+^ | 0.057^+^ | 0.057^+^ | 0.057^+^ | 0.057^+^ | 0.057^*^ |
|  |  | (0.037) | (0.031) | (0.031) | (0.032) | (0.031) | (0.024) |
| *18-29 (Ref. cat.)* |  | - | - | - | - | - | - |
|  |  |  |  |  |  |  |  |
| 30-39 |  | -0.009 | -0.020^+^ | -0.020^+^ | -0.024^+^ | -0.024^+^ | -0.024 |
|  |  | (0.015) | (0.012) | (0.012) | (0.013) | (0.013) | (0.016) |
| 40-49 |  | 0.013 | 0.025^**^ | 0.025^**^ | 0.022^*^ | 0.022^*^ | 0.022 |
|  |  | (0.012) | (0.009) | (0.009) | (0.009) | (0.009) | (0.015) |
| 50+ |  | 0.042^*^ | 0.035^*^ | 0.035^*^ | 0.030^*^ | 0.030^*^ | 0.030^*^ |
|  |  | (0.019) | (0.016) | (0.016) | (0.013) | (0.014) | (0.015) |
| Personal normative beliefs |  |  | 0.090^**^ | 0.090^**^ | 0.100^**^ | 0.100^**^ | 0.100^**^ |
|  |  |  | (0.034) | (0.034) | (0.034) | (0.034) | (0.036) |
| Normative expectations |  |  | 0.659^***^ | 0.659^***^ | 0.648^***^ | 0.649^***^ | 0.649^***^ |
|  |  |  | (0.033) | (0.033) | (0.033) | (0.033) | (0.031) |
| Reward |  |  | 0.023 | 0.023 | 0.026^+^ | 0.026 | 0.026^+^ |
|  |  |  | (0.016) | (0.016) | (0.016) | (0.016) | (0.015) |
| Punish |  |  | 0.029^+^ | 0.029^+^ | 0.032^+^ | 0.033^+^ | 0.033^+^ |
|  |  |  | (0.017) | (0.017) | (0.017) | (0.017) | (0.019) |
| GDP per capita |  |  | -0.000 | -0.000 | -0.000 | -0.000 | -0.000 |
|  |  |  | (0.000) | (0.000) | (0.000) | (0.000) | (0.000) |
| Stringency Index |  |  |  |  | 0.000 | 0.000 | 0.000 |
|  |  |  |  |  | (0.001) | (0.001) | (0.001) |
| *log*(Population size) |  |  |  |  |  | 0.006 | 0.006 |
|  |  |  |  |  |  | (0.010) | (0.010) |
| Constant | 0.559^***^ | 0.534^***^ | -0.075 | -0.075 | -0.103 | -0.204 | -0.204 |
|  | (0.027) | (0.031) | (0.055) | (0.055) | (0.088) | (0.194) | (0.197) |
| SD(Constant) | 0.120^***^ | 0.115^***^ | 0.082^***^ | 0.082^***^ | 0.087^***^ | 0.085^***^ | 0.085^***^ |
|  | (0.021) | (0.021) | (0.019) | (0.019) | (0.021) | (0.022) | (0.016) |
| SD(Residual) | 0.214^***^ | 0.212^***^ | 0.173^***^ | 0.173^***^ | 0.172^***^ | 0.172^***^ | 0.172^***^ |
|  | (0.012) | (0.011) | (0.006) | (0.006) | (0.005) | (0.005) | (0.004) |
| SD(Day) |  |  |  | 0.000 | 0.000 | 0.000 | 0.000^***^ |
|  |  |  |  | 0.000 | (0.000) | (0.000) | (0.000) |
| Observations (individuals) | 1229 | 1224 | 1212 | 1212 | 1142 | 1142 | 1142 |
| Observations (groups) | 77 | 77 | 74 | 74 | 71 | 71 | 71 |

Standard errors in parentheses. Models 1-6 have standard errors clustered at the country level. Model 7 does not cluster standard errors.

^+^ *p* < 0.10, ^*^ *p* < 0.05, ^**^ *p* < 0.01, ^***^ *p* < 0.001

**Table S13**. Normative expectations and day. Multilevel model including full sample (77, 74, or 71 countries, 2020).

|  | Model 1 | Model 2 | Model 3 | Model 4 | Model 5 | Model 6 | Model 7 |
| --- | --- | --- | --- | --- | --- | --- | --- |
| Day | 0.001^***^ | 0.001^**^ | 0.000^*^ | 0.000^*^ | 0.001^*^ | 0.001^**^ | 0.001^*^ |
|  | (0.000) | (0.000) | (0.000) | (0.000) | (0.000) | (0.000) | (0.000) |
| *Female (Ref. cat.)* |  | - | - | - | - | - | - |
|  |  |  |  |  |  |  |  |
| Male |  | -0.022 | -0.018 | -0.018 | -0.009 | -0.009 | -0.009 |
|  |  | (0.021) | (0.016) | (0.016) | (0.015) | (0.015) | (0.013) |
| Prefer not to say |  | 0.028 | 0.026 | 0.026 | 0.026 | 0.026 | 0.026 |
|  |  | (0.051) | (0.028) | (0.028) | (0.028) | (0.028) | (0.059) |
| *Doctor (Ref. cat.)* |  | - | - | - | - | - | - |
|  |  |  |  |  |  |  |  |
| Nurse/Midwife |  | 0.020^+^ | -0.014^+^ | -0.014^+^ | -0.010 | -0.011 | -0.011 |
|  |  | (0.011) | (0.008) | (0.008) | (0.007) | (0.007) | (0.011) |
| Other, clinically trained |  | 0.010 | -0.021^*^ | -0.021^*^ | -0.021^+^ | -0.021^+^ | -0.021 |
|  |  | (0.023) | (0.010) | (0.010) | (0.011) | (0.011) | (0.014) |
| Other, not clinically trained |  | 0.016 | -0.018 | -0.018 | -0.017 | -0.017 | -0.017 |
|  |  | (0.026) | (0.023) | (0.023) | (0.022) | (0.022) | (0.020) |
| *18-29 (Ref. cat.)* |  | - | - | - | - | - | - |
|  |  |  |  |  |  |  |  |
| 30-39 |  | 0.001 | 0.007 | 0.007 | 0.009 | 0.009 | 0.009 |
|  |  | (0.011) | (0.008) | (0.008) | (0.008) | (0.008) | (0.013) |
| 40-49 |  | -0.021^*^ | -0.026^***^ | -0.026^***^ | -0.025^**^ | -0.026^**^ | -0.026^*^ |
|  |  | (0.010) | (0.008) | (0.008) | (0.008) | (0.008) | (0.012) |
| 50+ |  | 0.005 | -0.019^+^ | -0.019^+^ | -0.022^+^ | -0.022^+^ | -0.022^+^ |
|  |  | (0.011) | (0.010) | (0.010) | (0.012) | (0.012) | (0.012) |
| Personal normative beliefs |  |  | 0.203^***^ | 0.203^***^ | 0.203^***^ | 0.202^***^ | 0.202^***^ |
|  |  |  | (0.030) | (0.030) | (0.030) | (0.030) | (0.029) |
| Empirical expectations |  |  | 0.448^***^ | 0.448^***^ | 0.439^***^ | 0.440^***^ | 0.440^***^ |
|  |  |  | (0.040) | (0.040) | (0.041) | (0.041) | (0.020) |
| Reward |  |  | 0.023^*^ | 0.023^*^ | 0.025^*^ | 0.025^*^ | 0.025^*^ |
|  |  |  | (0.011) | (0.011) | (0.012) | (0.012) | (0.012) |
| Punish |  |  | 0.003 | 0.003 | 0.001 | 0.000 | 0.000 |
|  |  |  | (0.016) | (0.016) | (0.016) | (0.016) | (0.016) |
| GDP per capita |  |  | 0.000^*^ | 0.000^*^ | 0.000^**^ | 0.000^**^ | 0.000^**^ |
|  |  |  | (0.000) | (0.000) | (0.000) | (0.000) | (0.000) |
| Stringency Index |  |  |  |  | 0.002^**^ | 0.002^**^ | 0.002^**^ |
|  |  |  |  |  | (0.001) | (0.001) | (0.001) |
| *log*(Population size) |  |  |  |  |  | -0.003 | -0.003 |
|  |  |  |  |  |  | (0.006) | (0.006) |
| Constant | 0.758^***^ | 0.764^***^ | 0.292^***^ | 0.292^***^ | 0.147^**^ | 0.207^+^ | 0.207^+^ |
|  | (0.021) | (0.023) | (0.029) | (0.029) | (0.055) | (0.106) | (0.125) |
| SD(Constant) | 0.091^***^ | 0.088^***^ | 0.040^***^ | 0.040^***^ | 0.042^***^ | 0.040^***^ | 0.040^***^ |
|  | (0.019) | (0.019) | (0.020) | (0.020) | (0.020) | (0.021) | (0.015) |
| SD(Residual) | 0.181^***^ | 0.181^***^ | 0.144^***^ | 0.144^***^ | 0.142^***^ | 0.142^***^ | 0.142^***^ |
|  | (0.012) | (0.013) | (0.008) | (0.008) | (0.008) | (0.008) | (0.003) |
| SD(Day) |  |  |  | 0.000 | 0.000 | 0.000 | 0.000^***^ |
|  |  |  |  | (0.000) | (0.000) | (0.000) | (0.000) |
| Observations (individuals) | 1235 | 1224 | 1212 | 1212 | 1142 | 1142 | 1142 |
| Observations (groups) | 77 | 77 | 74 | 74 | 71 | 71 | 71 |

Standard errors in parentheses. Models 1-6 use standard errors clustered at the country level. Model 7 does not cluster standard errors.

^+^ *p* < 0.10, ^*^ *p* < 0.05, ^**^ *p* < 0.01, ^***^ *p* < 0.001

**Table S14**. Empirical expectations and date. Multilevel model outputs excluding Kazakhstan (76 or 73 countries, 2020).

|  | Model 1 | Model 2 | Model 3 | Model 4 |
| --- | --- | --- | --- | --- |
| Day | 0.002^***^ | 0.001^**^ | 0.001^*^ | 0.001^*^ |
|  | (0.000) | (0.000) | (0.000) | (0.000) |
| *Female (Ref. cat.)* |  | - | - | - |
|  |  |  |  |  |
| Male |  | 0.007 | 0.024 | 0.024 |
|  |  | (0.021) | (0.016) | (0.016) |
| Prefer not to say |  | 0.037 | 0.045 | 0.045 |
|  |  | (0.057) | (0.036) | (0.036) |
| *Doctor (Ref. cat.)* |  | - | - | - |
|  |  |  |  |  |
| Nurse/Midwife |  | 0.060^*^ | 0.045^*^ | 0.045^*^ |
|  |  | (0.030) | (0.020) | (0.020) |
| Other, clinically trained |  | 0.090^*^ | 0.057^*^ | 0.057^*^ |
|  |  | (0.035) | (0.029) | (0.029) |
| Other, not clinically trained |  | 0.060 | 0.065 | 0.065 |
|  |  | (0.063) | (0.051) | (0.051) |
| *18-29 (Ref. cat.)* |  | - | - | - |
|  |  |  |  |  |
| 30-39 |  | -0.006 | -0.020 | -0.020 |
|  |  | (0.023) | (0.018) | (0.018) |
| 40-49 |  | 0.010 | 0.019 | 0.019 |
|  |  | (0.025) | (0.017) | (0.017) |
| 50+ |  | 0.071^**^ | 0.059^**^ | 0.059^**^ |
|  |  | (0.027) | (0.020) | (0.020) |
| Personal normative beliefs |  |  | 0.101^*^ | 0.101^*^ |
|  |  |  | (0.042) | (0.042) |
| Normative expectations |  |  | 0.696^***^ | 0.696^***^ |
|  |  |  | (0.035) | (0.035) |
| Reward |  |  | 0.008 | 0.008 |
|  |  |  | (0.023) | (0.023) |
| Punish |  |  | 0.020 | 0.020 |
|  |  |  | (0.029) | (0.029) |
| GDP per capita |  |  | -0.000 | -0.000 |
|  |  |  | (0.000) | (0.000) |
| Constant | 0.550^***^ | 0.513^***^ | -0.102^+^ | -0.102^+^ |
|  | (0.023) | (0.025) | (0.060) | (0.060) |
| SD(Constant) | 0.096^***^ | 0.094^***^ | 0.065^***^ | 0.065^***^ |
|  | (0.018) | (0.020) | (0.023) | (0.023) |
| SD(Residual) | 0.233^***^ | 0.231^***^ | 0.181^***^ | 0.181^***^ |
|  | (0.005) | (0.005) | (0.005) | (0.005) |
| SD(Day) |  |  |  | 0.000 |
|  |  |  |  | (0.000) |
| Observations (individuals) | 699 | 692 | 685 | 685 |
| Observations (groups) | 76 | 76 | 73 | 73 |

Standard errors in parentheses

^+^ *p* < 0.10, ^*^ *p* < 0.05, ^**^ *p* < 0.01, ^***^ *p* < 0.001

**Table S15**. Normative expectations and day. Multilevel model excluding Kazakhstan (76 or 73 countries, 2020).

|  | Model 1 | Model 2 | Model 3 | Model 4 |
| --- | --- | --- | --- | --- |
| Day | 0.001^***^ | 0.001^**^ | 0.000^*^ | 0.000^*^ |
|  | (0.000) | (0.000) | (0.000) | (0.000) |
| *Female (Ref. cat.)* |  | - | - | - |
|  |  |  |  |  |
| Male |  | -0.025 | -0.023 | -0.023 |
|  |  | (0.026) | (0.019) | (0.019) |
| Prefer not to say |  | -0.058 | -0.019 | -0.019 |
|  |  | (0.116) | (0.066) | (0.066) |
| *Doctor (Ref. cat.)* |  | - | - | - |
|  |  |  |  |  |
| Nurse/Midwife |  | 0.018 | -0.025^+^ | -0.025^+^ |
|  |  | (0.022) | (0.014) | (0.014) |
| Other, clinically trained |  | 0.041^+^ | -0.013 | -0.013 |
|  |  | (0.023) | (0.017) | (0.017) |
| Other, not clinically trained |  | -0.012 | -0.051^+^ | -0.051^+^ |
|  |  | (0.041) | (0.028) | (0.028) |
| *18-29 (Ref. cat.)* |  | - | - | - |
|  |  |  |  |  |
| 30-39 |  | 0.007 | 0.011 | 0.011 |
|  |  | (0.019) | (0.014) | (0.014) |
| 40-49 |  | -0.011 | -0.017 | -0.017 |
|  |  | (0.019) | (0.014) | (0.014) |
| 50+ |  | 0.013 | -0.029 | -0.029 |
|  |  | (0.025) | (0.019) | (0.019) |
| Personal normative beliefs |  |  | 0.171^***^ | 0.171^***^ |
|  |  |  | (0.035) | (0.035) |
| Empirical expectations |  |  | 0.494^***^ | 0.494^***^ |
|  |  |  | (0.041) | (0.041) |
| Reward |  |  | 0.031^+^ | 0.031^+^ |
|  |  |  | (0.016) | (0.016) |
| Punish |  |  | 0.023 | 0.023 |
|  |  |  | (0.022) | (0.022) |
| GDP per capita |  |  | 0.000^**^ | 0.000^**^ |
|  |  |  | (0.000) | (0.000) |
| Constant | 0.754^***^ | 0.751^***^ | 0.277^***^ | 0.277^***^ |
|  | (0.020) | (0.020) | (0.031) | (0.031) |
| SD(Constant) | 0.073^***^ | 0.066^***^ | 0.023^***^ | 0.023^***^ |
|  | (0.014) | (0.015) | (0.024) | (0.024) |
| SD(Residual) | 0.199^***^ | 0.200^***^ | 0.155^***^ | 0.155^***^ |
|  | (0.011) | (0.010) | (0.007) | (0.007) |
| SD(Day) |  |  |  | 0.000 |
|  |  |  |  | (0.000) |
| Observations (individuals) | 697 | 690 | 685 | 685 |
| Observations (groups) | 76 | 76 | 73 | 73 |

Standard errors in parentheses

^+^ *p* < 0.10, ^*^ *p* < 0.05, ^**^ *p* < 0.01, ^***^ *p* < 0.001

**Table S16**. Empirical expectations and date. Multilevel model outputs excluding Great Britain (76 or 73 countries, 2020).

|  | Model 1 | Model 2 | Model 3 | Model 4 |
| --- | --- | --- | --- | --- |
| Day | 0.001^**^ | 0.001^**^ | 0.001^*^ | 0.001^*^ |
|  | (0.000) | (0.000) | (0.000) | (0.000) |
| *Female (Ref. cat.)* |  | - | - | - |
|  |  |  |  |  |
| Male |  | -0.012 | 0.014 | 0.014 |
|  |  | (0.020) | (0.018) | (0.018) |
| Prefer not to say |  | 0.026 | 0.023 | 0.023 |
|  |  | (0.028) | (0.019) | (0.019) |
| *Doctor (Ref. cat.)* |  | - | - | - |
|  |  |  |  |  |
| Nurse/Midwife |  | 0.055^***^ | 0.045^***^ | 0.045^***^ |
|  |  | (0.015) | (0.012) | (0.012) |
| Other, clinically trained |  | 0.059^**^ | 0.050^**^ | 0.050^**^ |
|  |  | (0.023) | (0.019) | (0.019) |
| Other, not clinically trained |  | 0.042 | 0.034 | 0.034 |
|  |  | (0.034) | (0.025) | (0.025) |
| *18-29 (Ref. cat.)* |  | - | - | - |
|  |  |  |  |  |
| 30-39 |  | -0.003 | -0.018 | -0.018 |
|  |  | (0.015) | (0.013) | (0.013) |
| 40-49 |  | 0.009 | 0.023^*^ | 0.023^*^ |
|  |  | (0.013) | (0.011) | (0.011) |
| 50+ |  | 0.038^+^ | 0.030^*^ | 0.030^*^ |
|  |  | (0.020) | (0.014) | (0.014) |
| Personal normative beliefs |  |  | 0.081^*^ | 0.081^*^ |
|  |  |  | (0.035) | (0.035) |
| Normative expectations |  |  | 0.658^***^ | 0.658^***^ |
|  |  |  | (0.036) | (0.036) |
| Reward |  |  | 0.016 | 0.016 |
|  |  |  | (0.018) | (0.018) |
| Punish |  |  | 0.019 | 0.019 |
|  |  |  | (0.017) | (0.017) |
| GDP per capita |  |  | -0.000 | -0.000 |
|  |  |  | (0.000) | (0.000) |
| Constant | 0.570^***^ | 0.548^***^ | -0.045 | -0.045 |
|  | (0.027) | (0.030) | (0.048) | (0.048) |
| SD(Constant) | 0.121^***^ | 0.117^***^ | 0.085^***^ | 0.085^***^ |
|  | (0.021) | (0.021) | (0.019) | (0.019) |
| SD(Residual) | 0.212^***^ | 0.211^***^ | 0.171^***^ | 0.171^***^ |
|  | (0.012) | (0.012) | (0.005) | (0.005) |
| SD(Day) |  |  |  | 0.000 |
|  |  |  |  | (0.000) |
| Observations (individuals) | 1107 | 1102 | 1090 | 1090 |
| Observations (groups) | 76 | 76 | 73 | 73 |

Standard errors in parentheses

^+^ *p* < 0.10, ^*^ *p* < 0.05, ^**^ *p* < 0.01, ^***^ *p* < 0.001

**Table S17**. Normative expectations and day. Multilevel model excluding Great Britain (76 or 73 countries, 2020).

|  | Model 1 | Model 2 | Model 3 | Model 4 |
| --- | --- | --- | --- | --- |
| Day | 0.001^**^ | 0.001^*^ | 0.000^*^ | 0.000^*^ |
|  | (0.000) | (0.000) | (0.000) | (0.000) |
| *Female (Ref. cat.)* |  | - | - | - |
|  |  |  |  |  |
| Male |  | -0.034^+^ | -0.025 | -0.025 |
|  |  | (0.020) | (0.016) | (0.016) |
| Prefer not to say |  | 0.005 | 0.010 | 0.010 |
|  |  | (0.066) | (0.032) | (0.032) |
| *Doctor (Ref. cat.)* |  | - | - | - |
|  |  |  |  |  |
| Nurse/Midwife |  | 0.015 | -0.016^+^ | -0.016^+^ |
|  |  | (0.011) | (0.009) | (0.009) |
| Other, clinically trained |  | 0.007 | -0.016 | -0.016 |
|  |  | (0.026) | (0.013) | (0.013) |
| Other, not clinically trained |  | 0.016 | -0.010 | -0.010 |
|  |  | (0.028) | (0.020) | (0.020) |
| *18-29 (Ref. cat.)* |  | - | - | - |
|  |  |  |  |  |
| 30-39 |  | 0.007 | 0.009 | 0.009 |
|  |  | (0.012) | (0.009) | (0.009) |
| 40-49 |  | -0.023^*^ | -0.027^**^ | -0.027^**^ |
|  |  | (0.011) | (0.009) | (0.009) |
| 50+ |  | 0.009 | -0.014 | -0.014 |
|  |  | (0.013) | (0.009) | (0.009) |
| Personal normative beliefs |  |  | 0.197^***^ | 0.197^***^ |
|  |  |  | (0.033) | (0.033) |
| Empirical expectations |  |  | 0.460^***^ | 0.460^***^ |
|  |  |  | (0.046) | (0.046) |
| Reward |  |  | 0.026^*^ | 0.026^*^ |
|  |  |  | (0.013) | (0.013) |
| Punish |  |  | 0.000 | 0.000 |
|  |  |  | (0.017) | (0.017) |
| GDP per capita |  |  | 0.000^*^ | 0.000^*^ |
|  |  |  | (0.000) | (0.000) |
| Constant | 0.756^***^ | 0.769^***^ | 0.289^***^ | 0.289^***^ |
|  | (0.023) | (0.026) | (0.034) | (0.034) |
| SD(Constant) | 0.090^***^ | 0.087^***^ | 0.041^***^ | 0.041^***^ |
|  | (0.020) | (0.020) | (0.021) | (0.021) |
| SD(Residual) | 0.182^***^ | 0.182^***^ | 0.145^***^ | 0.145^***^ |
|  | (0.014) | (0.014) | (0.009) | (0.009) |
| SD(Day) |  |  |  | 0.000 |
|  |  |  |  | (0.000) |
| Observations (individuals) | 1107 | 1102 | 1090 | 1090 |
| Observations (groups) | 76 | 76 | 73 | 73 |

Standard errors in parentheses

^+^ *p* < 0.10, ^*^ *p* < 0.05, ^**^ *p* < 0.01, ^***^ *p* < 0.001

**Table S18**. Empirical expectations and COVID-19 cases. Multilevel model outputs including full sample (77 countries, 2020).

|  | Model 1 | Model 2 |
| --- | --- | --- |
| Total COVID-19 cases | 0.000^***^ |  |
|  | (0.000) |  |
| Total COVID-19 cases/million population |  | 0.000^**^ |
|  |  | (0.000) |
| Constant | 0.621^***^ | 0.603^***^ |
|  | (0.019) | (0.021) |
| SD(Constant) | 0.114^***^ | 0.118^***^ |
|  | (0.021) | (0.022) |
| SD(Residual) | 0.216^***^ | 0.215^***^ |
|  | (0.012) | (0.012) |
| Observations (individuals) | 1229 | 1229 |
| Observations (groups) | 77 | 77 |

Standard errors in parentheses. Standard errors clustered at the country level.

^+^ *p* < 0.10, ^*^ *p* < 0.05, ^**^ *p* < 0.01, ^***^ *p* < 0.001

**Table S19**. Normative expectations and COVID-19 cases. Multilevel model outputs including full sample (77 countries, 2020).

|  | Model 1 | Model 2 |
| --- | --- | --- |
| Total COVID-19 cases | 0.000^**^ |  |
|  | (0.000) |  |
| Total COVID-19 cases/million population |  | 0.000^**^ |
|  |  | (0.000) |
| Constant | 0.798^***^ | 0.787^***^ |
|  | (0.015) | (0.017) |
| SD(Constant) | 0.088^***^ | 0.087^***^ |
|  | (0.020) | (0.020) |
| SD(Residual) | 0.182^***^ | 0.181^***^ |
|  | (0.013) | (0.013) |
| Observations (individuals) | 1229 | 1229 |
| Observations (groups) | 77 | 77 |

Standard errors in parentheses. Standard errors clustered at the country level.

^+^ *p* < 0.10, ^*^ *p* < 0.05, ^**^ *p* < 0.01, ^***^ *p* < 0.001

# Supplementary Figures

**Figure S1**. Full sample: histograms of personal normative beliefs, social expectations (NE, EE, EE busy, EE senior), punishment, and reward (77 or 76 countries, 2020).

**Figure S2**. Sample excluding Kazakhstan: histograms of personal normative beliefs, social expectations (NE, EE, EE busy, EE senior), punishment, and reward (76 or 75 countries, 2020).

**Figure S3**. Sample excluding Great Britain: histograms of personal normative beliefs, social expectations (NE, EE, EE busy, EE senior), punishment, and reward (76 or 75 countries, 2020).

**Figure S4**. Social expectations and personal normative beliefs according to occupation, age, and gender. Kazakhstan excluded (76 countries, 2020).

**Figure S5**. Social expectations and personal normative beliefs according to occupation, age, and gender. Great Britain excluded (76 countries, 2020).

**Figure S6**. Reward and punishment according to occupation, age, and gender. Kazakhstan excluded (76 or 75 countries, 2020).

**Figure S7**. Reward and punishment according to occupation, age, and gender. Great Britain excluded (76 or 75 countries, 2020).

# References

1. Semaan A, Audet C, Huysmans E, Afolabi B, Assarag B, Banke-Thomas A, et al. Voices from the frontline: findings from a thematic analysis of a rapid online global survey of maternal and newborn health professionals facing the COVID-19 pandemic. BMJ Global Health. 2020 Jun 1;5(6):e002967.
